# Effectuality study of a 3D motion correction algorithm in C-arm CTs of severely impaired image quality during transarterial chemoembolization

**DOI:** 10.1186/s40644-022-00473-3

**Published:** 2022-07-30

**Authors:** Lena S. Becker, Cornelia L. A. Dewald, Christian von Falck, Thomas Werncke, Sabine K. Maschke, Roman Kloeckner, Frank K. Wacker, Bernhard C. Meyer, Jan B. Hinrichs

**Affiliations:** 1 Department of Diagnostic and Interventional Radiology, Hannover Medical School, OE8220 Carl-Neuberg-Str. 1, 30625 Hannover, Germany; 2 Department of Diagnostic and Interventional Radiology, Johannes Gutenberg-University Medical Centre, Mainz, Germany

**Keywords:** C-Arm CT, Transarterial chemoembolization, Motion correction algorithm, Interventional Radiology

## Abstract

**Background:**

To evaluate effectivity of a 3D-motion correction algorithm in C-Arm CTs (CACT) with limited image quality (IQ) during transarterial chemoembolization (TACE).

**Methods:**

From 1/2015–5/2021, 644 CACTs were performed in patients during TACE. Of these, 27 CACTs in 26 patients (18 m, 8f; 69.7 years ± 10.7 SD) of limited IQ were included. Post-processing of the original raw-data sets (CACT_Org_) included application of a 3D-motion correction algorithm and bone segmentation (CACT_MC_no_bone_). Four radiologists (R1-4) compared the images by choosing their preferred dataset and recommending repeat acquisition in case of severe IQ-impairment. R1,2 performed additional grading of intrahepatic vessel visualization, presence/extent of movement artifacts, and overall IQ.

**Results:**

R1,2 demonstrated excellent interobserver agreement for overall IQ (ICC 0.79,*p* < 0.01) and the five-point vessel visualization scale before and after post-processing of the datasets (ICC 0.78,*p* < 0.01). Post-processing caused significant improvement, with overall IQ improving from 2.63 (CACT_Org_) to 1.39 (CACT_MC_no_bone_;*p* < 0.01) and a decrease in the mean distance of identifiable, subcapsular vessels to the liver capsule by 4 mm (*p* < 0.01). This proved especially true for datasets with low parenchymal and high hepatic artery contrast. A good interobserver agreement (ICC = 0.73) was recorded concerning the presence of motion artifacts, with significantly less discernible motion after post-processing (CACT_Org_:1.31 ± 1.67, CACT_MC_no_bone_:1.00 ± 1.34, *p* < 0.01). Of the 27 datasets, ≥ 23 CACT_MC_no_bone_ were preferred, with identical datasets chosen by the readers to show benefit from the algorithm.

**Conclusion:**

Application of a 3D-motion correction algorithm significantly improved IQ in diagnostically limited CACTs during TACE, with the potential to decrease repeat acquisitions.

## Background

Multiple interventions profit from C-arm computed tomography (CACT) as a guidance tool [1–11]. Improved soft-tissue resolution as well as elimination of vessel superposition with resulting high-precision lesion detection and sophisticated catheter navigation represent important attributes that have led to studies reporting advantages of CACT over 2D digital subtraction angiography (DSA) [1–5, 12–18]. A meta-analysis of TACE interventions in hepatocellular carcinoma (HCC) reported an increase in sensitivity of up to 38% in favor of CACT concerning detection of tumors and tumor feeding arteries [6]. With radiation exposure during CACT being sizeable, its use may be considered as a preliminary investment for optimal feeder detection and navigational planning. However, this investment can be rendered pointless by insufficient image quality (IQ), originating predominantly from the method’s vulnerability towards motion. Motion artifacts due to patient movement on the angiography table, breathing or cardiac pulsation may cause loss of critical periprocedural information as well as severely compromise the interventional outcome [1–5]. Based on a vascular reconstruction algorithm (CAVAREC, Siemens Healthcare, Forchheim, Germany), initially developed for motion compensation in 3D cardiac imaging studies [19–21], its beneficial effect on diagnostic CACT datasets by evaluating both objective and subjective IQ criteria, has been previously described [22, 23]. While the mean dataset IQ in the database was serviceable, the current study focused on severely impaired CACT images to diminish any influence of a CACT’s good baseline image quality on the algorithm’s effectiveness and to emphasize the potential value of CACT post-processing methods in decreasing motion artefacts.

The aim of this retrospective single-center study was thus to evaluate the feasibility and effectiveness of a motion-compensating, 3D reconstruction technique on CACTs with substantially impaired image quality and to identify determinant factors for its effectiveness.

## Methods

**Fig. 1:**
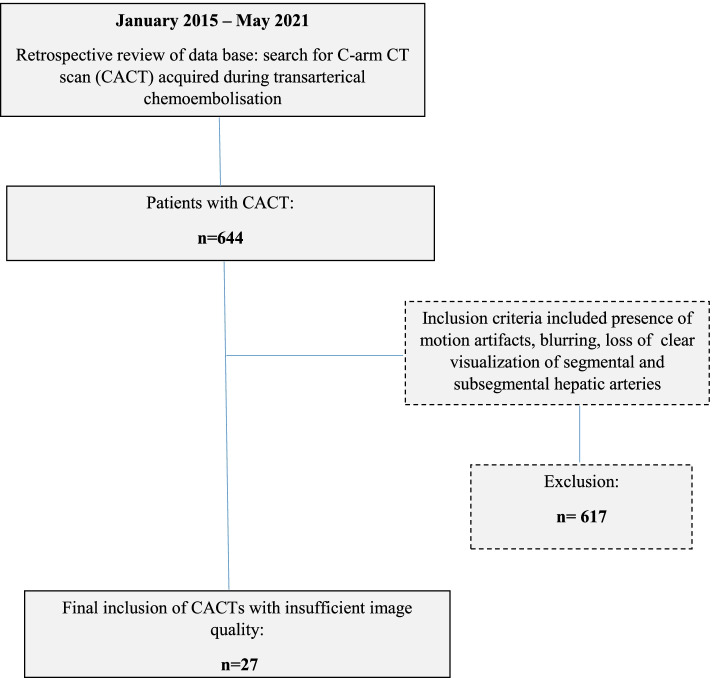
Flow chart of patient selection in the present study

Our hospital’s Institutional Review Board (Ethics committee Hannover Medical School; Nr. 8316_BO_K_2019) approved this retrospective study. All patients gave written consent. The indication for TACE was obtained by an inter-disciplinary tumor board. All TACE procedures from 1/2015 to 5/2021 (*n* = 644) were retrospectively reviewed. Based on previously published work determining influencing factors on image quality in CACTs as well as the IQ-improving effect of the 3D motion compensating algorithm, we purposefully selected datasets with poor image quality as main inclusion requirement (see Fig. 1), represented by substantially limited visualization of both central as well as peripheral hepatic arteries, including distinct blurriness of vessel margins and/ or severe cardiorespiratory motion artifacts [22, 23]. The study population thus comprised of 27 CACTs in 26 patients (18 m, 8 f; mean age: 69.7 years ± 10.7 SD)– one patient having received a CACT of both the left and the right liver artery due to variant hepatic anatomy—with patient characteristics shown in Table 1.

**Table 1:** Patient demographics and image quality data

Age (years)	69.7 ± 10.7	
Gender		
Female (%)	8 (30.8)	
Male (%)	18 (69.2)	
CACT before TACE intervention		
Included	39	
Excluded (catheter dislocation, moderate IQ)	13	
Bad IQ due to (several may apply):		
Cardiorespiratory motion artifacts	16	
Low hepatic artery-to-parenchyma enhancement ratio	14	
3D motion recompensation algorithm		
Technical feasibility	27	
Image preference concerning TACE intervention		
	**R 1, 2**	**R 3,4**
CACT_MC_no bone_ > CACT_Org_	22	19
CACT_MC_no bone_ = CACT_Org_	/	5
CACT_MC_no bone_ < CACT_Org_	**5**	**3**

**Abbreviations:** *CACT*_*Org*_ original C-Arm CT, *CACT*_*MC no bone*_ C-arm CT after motion correction and bone segmentation, *IQ* Image quality, *TACE* transarterial chemoembolization

### TACE procedure

Ultrasound-guided assessment of the femoral artery was performed after application of local anesthesia. Mesenterico-portography, sequential hepatic angiography and a CACT were acquired to assess vascular anatomy, to analyze tumor-feeding arteries and to plan an adequate, supraselective catheter position for treatment. The arms were elevated to avoid streaking artifacts. Patients were instructed to hold their breath for 10 s, which—unlike most patients in every-day practice—the majority of patients included in this study was not able to perform sufficiently, causing breathing motion artifacts (*n* = 16) of different proportions. CACT was obtained using the manufacturer’s preset (Artis Q: 6 s DR DynaCT, acquisition time of 6 s, fixed-tube-detector distance of 0.9 m, total acquisition angle of 200°, projection increment of 0.5°, 396 projectional images, 1 k-matrix, zoom factor 0, FOV 48 cm, system-detector dose per image of 0.36 lGy; Artis pheno: 5 s DR DynaCT, acquisition time of 5 s, fixed-tube-detector distance of 0.9 m, total acquisition angle of 200°, projection increment of 0.5°, 396 projectional images, 1 k-matrix, zoom factor 0, FOV 52 cm, system-detector dose per image of 0.36 lGy). Contrast medium was injected in accordance with our standard protocol for diagnostic catheter (*n* = 15, flow rate 5 ml/sec) or microcatheter injections (*n* = 12, flow rate 2.5 ml/sec). Doxorubicin-loaded drug-eluting beads of 30–60 um size (HepaSphere®, Merit Medical Europe, Maastricht, Netherlands) were injected into the tumor-feeding artery via the microcatheter (Merit Maestro™, Tenor™ 0.014 guidewire, Merit Medical Systems, Utah, USA), with stasis in the tumor-feeding arteries delineating the endpoint of the intervention.

### Imaging and post-processing

All TACE procedures were performed by board-certified interventional radiologists at our institution on either a monoplane, ceiling-mounted or monoplane, robotic-arm-mounted angiographic system (Artis Q®, ARTIS pheno®, Siemens Healthcare, Forchheim, Germany) as available during clinical routine. Image acquisition commenced simultaneously to contrast injection, with the C-arm mounted X-ray source and detector rotating around the patient on a circular trajectory. The 3D reconstruction prototype software, developed and modified by the manufacturer (Siemens Healthcare, Forchheim, Germany), was installed on a dedicated workstation (syngo X Workplace®, Software Version VD20C, Siemens Healthcare, Forchheim, Germany) and applied to the original raw datasets. These were retrospectively modified by the algorithm’s utilization of iterative motion estimation and compensation of a 4D deformable motion, as previously published elsewhere [19–23]. For motion correction of selective CACT images of the hepatic arteries, we used 500 iterations in total and an iteration update/display every 100 iterations. The step size was 2 mm and motion resolution 25 mm. Manual volume punching of stationary high-contrast objects such as bones or extraneous materials was performed by a blinded radiologist (SKM), as these could lead to potential falsifications of motion correction in the liver. The aforementioned post-processing, enabled quantitative and qualitative comparisons between two datasets: the original CACT (CACT_Org_) and the CACT after motion correction and bone removal (CACT_MC_no bone_).

### Image interpretation and analysis

Two radiologists of 10 and 3 years of experience (JBH, LSB: reader 1,2), blinded to the nature of the data set (CACT_Org_ vs. CACT_MC_no bone_), performed side-by-side grading of randomly assigned images before and after the application of the motion compensating algorithm, with concern to the following categories: 1) overall image quality (grade 1–3: good, moderate, poor), 2) vessel visualization and sharpness (i.e., clear visualization of all hepatic arteries, including subsegmental and subcapsular branches, grade 1–5), presence of artifacts (preponderantly induced by breathing or cardiac motion), and their image preference for TACE intervention (for details see Table 2).

**Table 2:** Summary of CACT IQ evaluation criteria

Overall IQ	
*Grading*	1: Good
	2 Moderate
	3: Poor
Vessel visualization	
*Grading*	1: clear visualization of all hepatic arteries including fine peripheral hepatic arteries at the subcapsular region without blurring
	2: clear visualization of hepatic arteries up to subsegmental level without blurring, but indisctinct fine peripheral hepatic arteries at the subcapsular region
	3: blurriness of hepatic arteries but traceable up to subsegmental level
	4: moderate blurring of hepatic arteries with pruning of subsegmental arteries
	5: severe blurring of hepatic arteries with difficulty in tracing segmental hepatic arteries
Presence of (cardiorespiratory) artifacts	
- Diaphragmatic motion d	Definition: Motion artifact width of right hemidiaphragm on sagittal image
*Grading*	0: none (< 0.1 mm)
	1: mild (0.1- 2 mm)
	2: moderate (2.1–3.5 mm)
	3:severe (> 3.5 mm)
Hepatic artery-to-parenchyma ratio	Definition: Ratio of intraarterial HU value 2 cm distally to the catheter tip (ROI fitted to two thirds of the vessel diameter) and maximal parenchymal enhancement
Preferred dataset for TACE	CACT_Org_ vs. CACT_MC_no bone_
Need for additional imaging	Yes/no

**Abbreviations:** *CACT*_*Org*_ original C-Arm CT, *CACT*_*MC_no bone*_C-arm CT after motion correction and bone segmentation, *HU* Hounsfield Units, *IQ* Image quality, *ROI* region of interest, *TACE* transarterial chemoembolization

To further validate the algorithm’s effect and to reproduce a realistic clinical setting, both the original as well as the post-processed datasets were additionally offered to two blinded radiologists of 11 and 4 years of experience (CvF, CLAD: reader 3,4), to document their preferred dataset for a transarterial chemoembolization of the liver, their recommendation for additional imaging (e.g., repeat CACT), and to compare the datasets by using a five-point Likert scale.

To quantitatively compare the hepatic artery delineation on both cone-beam CT datasets, the furthest peripheral position with preserved demarcation of the contrast-enhanced vessel lumen within the hepatic artery branch of each segment to the liver capsule was determined in a thin, axial multiplanar reformation (1 mm in both modalities; JBH and LSB in consensus). Additional measurements of intraarterial enhancement in the common, right or left hepatic artery as well as in areas of maximum liver parenchyma enhancement were performed (JBH and LSB in consensus), creating a ratio of the contrast in the liver arteries to the contrast in the liver parenchyma. For intravascular contrast quantification, a circular region of interest, fitted to at least two thirds of the vessel diameter and located 2 cm distally of the catheter tip in a thin-sliced, coronal reformat. For image assessment, all readers were able to use thin-sliced multiplanar reformats (slice thickness B 0.49 mm) in axial, coronal, sagittal or oblique orientation and maximum intensity projections (MIP) on a 3D PACS workstation (Visage 7.1, Visage Imaging, Berlin, Germany).

### Statistical analysis

Descriptive statistical analyses of the patient’s demographic and angiographic data are presented as mean values ± standard deviation (sd). Performance of a Shapiro–Wilk test showed a normal distribution of values (*p* > 0.05). A two-sided t-test was performed to assess the differences between CACT_Org_ and CACT_MC_no bone_ by measuring the distance of delineated subcapsular vessels in each liver segment to the capsule. For the comparison of image quality with regards to vessel visualization, presence and amount of movement artifacts as well as overall image quality, a paired Wilcoxon-test, an interrater and an intermodality agreement were calculated, using the two-way intra-class correlation coefficient with absolute agreement (ICC 2.1). This was also used to calculate interobserver agreement of the aforementioned two radiologists. Values ≥ 0.75 represented excellent agreement, those from 0.6–0.74 good, from 0.4–0.59 fair, and < 0.4 poor agreement [24].

Statistical analysis was conducted with R (version 3.6.1, http://www.r-project.org with package ‘‘IRR’’ version 0.84.1).

## Results

**Fig. 2:**
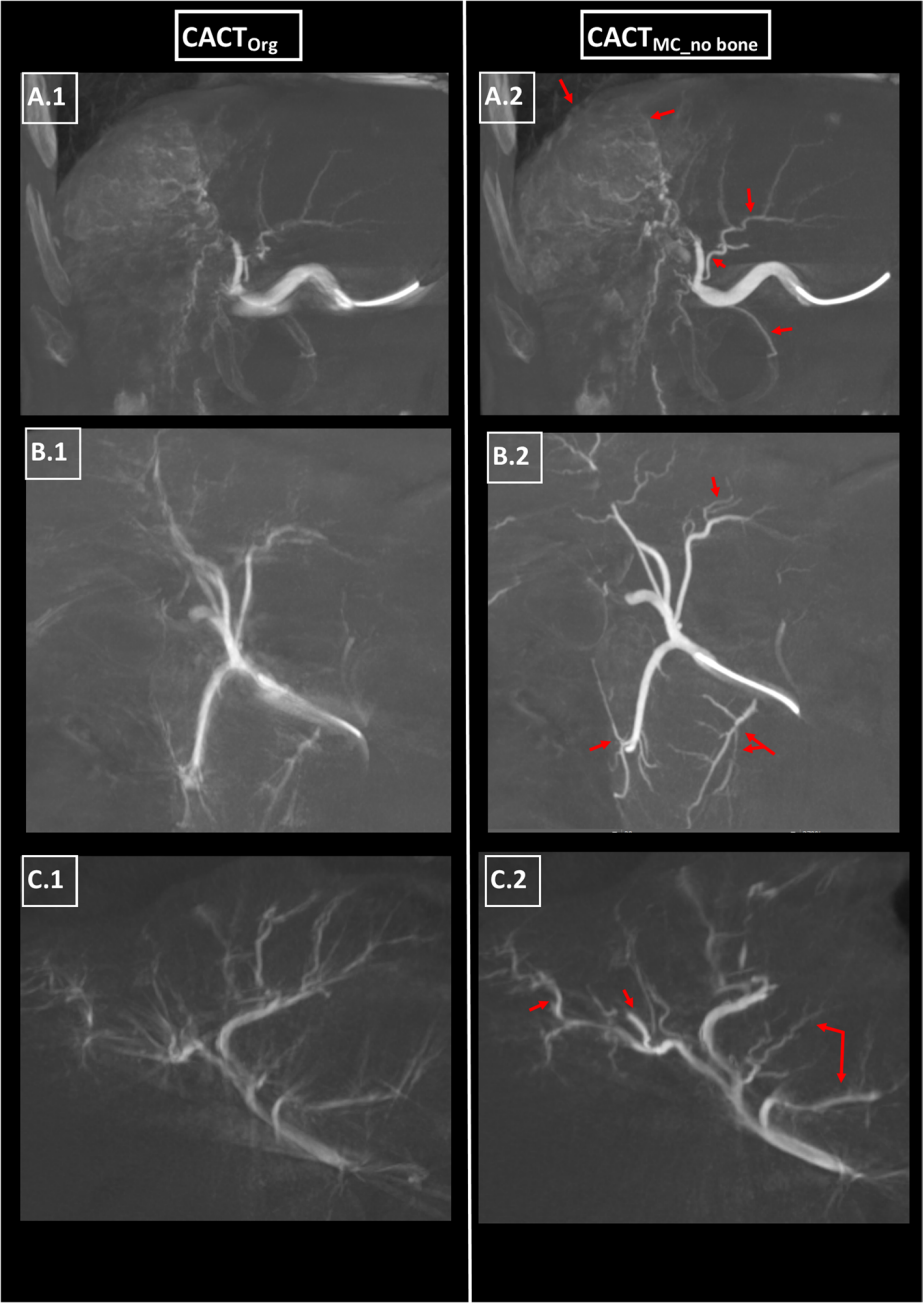
Three coronary 15 mm MIPs of the right (**A.1,2**), common (**B.1,2**) and left hepatic artery (**C.1**, **C2**) before and after application of the motion compensating algorithm (CACT_Org_ vs. CACT_MC_no bone_). **A.2** demonstrates a significantly less blurry central (right) hepatic artery, an increased number of vascular tumor feeders as potential embolization targets and a clear discrimination of the small arteries originating from the main stem when compared to **A.1**. **B.2** shows a significantly improved delineation of the common hepatic artery and its smaller branches, potentially benefitting their catherization. **C.2** shows improved visualization of the central (left) and peripheral hepatic arteries after application of the motion compensating algorithm and bone segmentation, though to a lesser degree peripherally than in **A**,**B**

**Fig. 3.**
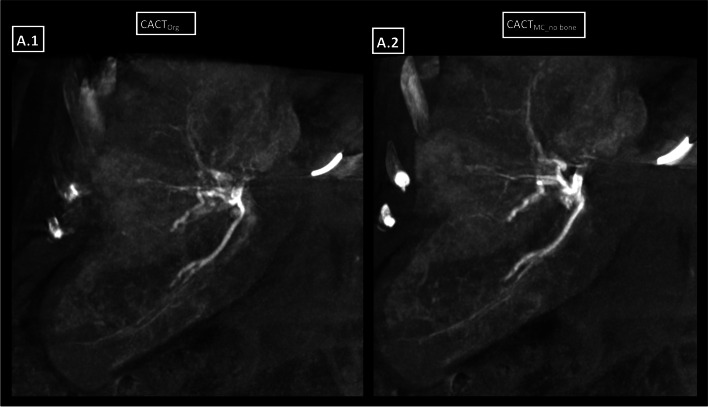
15 mm coronal MIP of a case with patchy enhancement of the liver parenchyma, and consecutively low ratio of hepatic artery-to-parenchyma contrast. There is no clear difference after application of the motion compensating algorithm, especially concerning vessel blurriness in the periphery

Successful application of the algorithm to the datasets proved feasible in all cases. Significantly better IQ as well as an excellent interobserver agreement (ICC: 0.79) could be demonstrated by comparing the blinded radiologists’ gradings of overall IQ (1: good – 3: poor) before and after algorithm application, with CACT_Org_ receiving a mean grade of 2.63 ± 0.7 (*p* < 0.01), whilst CACT_MC_no bone_ received a mean IQ value of 1.39 ± 0.8 (see also Fig. 2). Similar results were achieved by comparing the excellent consensus results of reader 1,2 (ICC: 0.78; CACT_Org_: 3.13 ± 1.07, CACT_MC_no bone_: 1.56 ± 1.01, *p* < 0.01). Of the 27 datasets, 23 CACT_MC_no bone_ were preferred to CACT_Org_, while both readers 1 and 2 chose the identical four datasets to not have profited from the motion correction algorithm (example see Fig. 3). Reader 1, a radiologist with 9 years of experience, recorded a recommendation of repeating two CACTs, reader 2 at two years of experience recommended four to be repeated. There was a good interobserver agreement (ICC 0.73) concerning the presence of (breathing) motion artifacts, with significantly more discernible motion in CACT_Org_ than after algorithm application (CACT_Org_: 1.31 ± 1.67, CACT_MC_no bone_: 1.00 ± 1.34, *p* < 0.01). Reader 2 detected fewer signs of motion artifacts than reader 1 in both datasets (R1: CACT_Org_: 1.48 ± 1.37, CACT_MC_no bone_: 1.26 ± 1.18, *p* < 0.01; R2: CACT_Org_: 1.15 ± 1.35, CACT_MC_no bone_: 0.74 ± 0.94, *p* < 0.01).

The other two radiologists (reader 3 and 4), representing the clinical setting, demonstrated clear preference for the CACT_MC_no bone_, with only three (identical) post-processed datasets being considered inferior to CACT_Org_. A significant, good interobserver agreement (ICC 0.74, *p* < 0.01) concerning the five-point Likert-scale comparison of the datasets resulted, with CACT_MC_no bone_ receiving a mean score of 1.55 (*p* < 0.01). While the radiologist with ten years of experience (reader 3) recommended repetition of four CACTs, reader 4 (three years of experience) advocated eight repetition scans. An overview of the interrater results is also given in Table 3.

**Table 3:** Tabellarized results from the interrater analysis

	**Reader 1**		**Reader 2**		**Reader 3**		**Reader 4**	
	**CACT**_**Org**_	**CACT**_**MC_no bone**_	**CACT**_**Org**_	**CACT**_**MC_no bone**_	**CACT**_**Org**_	**CACT**_**MC_no bone**_	**CACT**_**Org**_	**CACT**_**MC_no bone**_
**Vessel visualization** **(1–5)**	3.07 ± 0.78	1.61 ± 0.79	3.19 ± 0.74	1.45 ± 0.64	n.a		n.a	
	*Mean overall score (R1* + *R2):* CACT_Org_: 3.07 ± 0.75 CACT_MC_no bone_: 1.55 ± 0.72 * ICC = 0.74 *							
**Overall image quality (1–3)**	2.52 ± 0.51	1.41 ± 0.57	2.7 ± 0.45	1.37 ± 0.56	n.a		n.a	
	*Mean overall IQ (R1* + *R2):* CACT_Org_: 2.63 ± 0.7 CACT_MC_no bone_: 1.39 ± 0.8 * ICC = 0.79 *							
**Presence of breathing motiom artifacts (yes/ no)**	*n* = 15		*n* = 11		n.a		n.a	
	ICC 0.73 *							
**Preferred dataset**	*n* = 4	*n* = 20	*n* = 4	*n* = 20	*n* = 3	*n* = 21	*n* = 2	*n* = 22
**Extent of preference (-2: much less – + 2: much more)**					0.9		1.2	
**Need for additional/repeat imaging (yes/no)**	*n* = 2		*n* = 4		*n* = 4		*n* = 8	
**Mean distance of subcapsular, segmental vessels to liver capsule [mm]**	15.3 ± 14.9		11.5 ± 13.5		n.a		n.a	

Abbreviations: CACT_Org_: original C-Arm CT dataset, CACT_MC_no bone_: post-processed C-Arm CT (motion correction, bone segmentation)

^*^p < 0.01

**Fig. 4:**
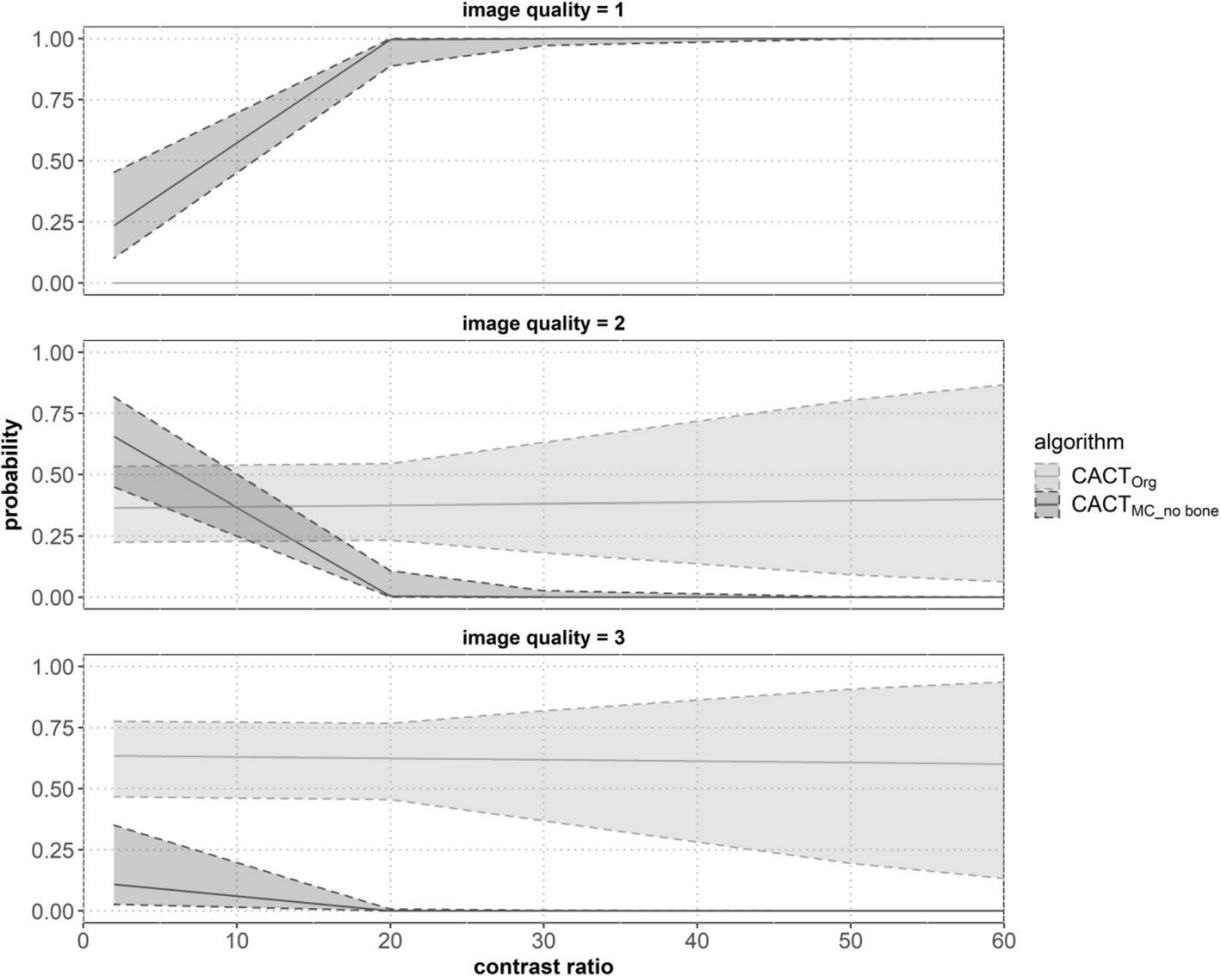
An ordinal logistic regression showed a significant correlation between the contrast ratio (CR) and the chosen image quality (IQ) of CACT_MC_no_bone_. This is reflected by the higher probability of choosing a better IQ with increasing contrast ratio and an odds ratio of 0.7 ± 1.1 (mean ± std. error). For CACT_Org_, no significant correlation was noted between the CR and the readers’ evaluation of IQ, which is also demonstrated by the comparable probabilities of choosing IQ and an odds ratio of 1 ± 1 (mean ± std error). The gray area between the perforated and continuous line represents the 95% confidence interval

The mean distance of clearly identifiable subcapsular vessels to the liver capsule lay at 15.3 ± 14.9 mm in CACT_Org_ (*p* < 0.01) compared to 11.5 ± 13.5 mm in CACT_MC_no bone_. This result could be duplicated not only for the liver as a whole but also for each individual segment. A linear regression model of the intraarterial and parenchymal density values demonstrated a significant interrelation between a high artery-to-parenchyma contrast ratio and effectiveness of the motion algorithm (see Fig. 4). This is reflected by the higher probability of choosing a better IQ with increasing contrast ratio and an odds ratio of 0.7 ± 1.1 (mean ± SD). For CACT_Org_, no significant correlation was noted between the CR and the readers’ evaluation of IQ, which is also demonstrated by the comparable probabilities of choosing IQ and an odds ratio of 1 ± 1 (mean ± SD). Measured in Hounsfield Units (HU), the mean ratio of datasets with high intraarterial contrast (2822 ± 1064.5 HU) and low parenchymal contrast (191.1 ± 95.86 HU), measured 17.9 ± 13.86. A low arterial contrast (1067.91 ± 416.38 HU) and relatively high and diffuse parenchymal (340.39 ± 151.51 HU) or even extrahepatic enhancement led to a low contrast ratio of 3.40 ± 1.18 probably influencing the algorithm negatively (Fig. 4).

## Discussion

The results of our study demonstrate that the application of a 3D motion compensating algorithm to CACT datasets in patients during TACE, with limited diagnostic image quality, could significantly improve overall IQ, vessel visualization both centrally and peripherally, and decrease the effect of motion artifacts. Use of such an algorithm might be able to save radiation exposure for patients and staff. This is especially important for younger radiologists who tended to recommend repetition of a CACT in up to 30% in our study.

The benefit of adjunct CACT versus stand-alone DSA is well documented for a variety of procedures, but especially for TACE [1–11], where improved soft-tissue resolution and elimination of vessel superposition helps to identify all tumor feeders thus supporting a more selective TACE. As the value of CACT largely depends on good image quality [6, 7, 25], the 3D-motion compensating image algorithm has shown to improve both, objective and subjective image quality criteria, in liver and lung, respectively [22, 23]. Having proven beneficial in optimizing sparse objects such as vessels, this algorithm includes manual volume punching for bone removal [19–23]. With only 27 of 644 inadequate CACTs in our CACT cohort, most patients seem to be able to perform the basic scan prerequisites of a 10 s breath-hold while laying motionless with their arms elevated above their heads. Hence, the majority of CACTs performed at our institution have adequate or good image quality. However, an inevitable factor such as cardiac motion, which in itself has been described to deteriorate CACT IQ in the left-sided liver segments especially, may be part of its source [25], a finding that could be duplicated by us. Especially in these cases of cardiorespiratory motion artifacts, simple enough post-processing may increase the tolerance for poor image quality and ultimately decrease the risks of a substantially compromised intervention or of having to repeat the imaging, especially as no new artifacts were documented by the use of the tool. For this reason, analysis of all CACT datasets from January 1^st^, 2015 – May 31^st^ 2021before TACE interventions was performed, in order to identify those that would especially stand to profit from IQ improvement due to being or bordering on being non-diagnostic. In addition to assessing overall IQ, we adapted previously published scoring criteria by Lee et al. [25] for CACT IQ and artifact quantification, by measuring the distance of clearly visualized subcapsular vessels to the capsule. The increase of peripherally identifiable subcapsular vessels could potentially improve software for embolization guidance, especially for peripheral lesions. While the majority of original CACT datasets improved significantly after application of the algorithm (see Fig. 3), few however did so on a lesser level or in one case, even decreased image perception (see Fig. 4). This did not correlate with the amount of motion artifacts perceived in the images nor with the type of catheter (micro- vs. standard diagnostic catheter) employed. By measuring both the intraarterial as well as the parenchymal hepatic contrast densities and calculating a hepatic artery-to-parenchyma-ratio, this led to the discovery of its significant correlation with algorithm effectuality. Measured in Hounsfield Units (HU) two centimeters distally to the catheter tip in the hepatic artery of CACT_Org_, high intraarterial contrast with mean values of roughly 2800 HU and low parenchymal contrast of 190 HU, measured in the epicenter of liver parenchyma enhancement, led to a high ratio of nearly 18. A low arterial contrast (1.000 HU) and relatively high and diffuse parenchymal (340 HU) or even extrahepatic enhancement, led to a low contrast ratio of 3. Enhancing tumor formations of different sizes did not interfere with the algorithm, however high extrahepatic (e.g., gastric, pancreatic) contrast enhancement in the three original datasets graded as superior to the post-processed CACTs, served as an indicator of a lower intrahepatic contrast dose and hence, an insufficient advantage from the algorithm. Guided by the maximum values of the unsuccessfully improved datasets (mean contrast ratio: 5.78) and the minimum of the successfully improved datasets (mean contrast ratio:7.32), a contrast ratio of at least 6–7 is needed for the algorithm to take effect and may thus be potentially used as an uncomplicated benchmark to roughly estimate its benefit. This may be of special help to less experienced interventionalists, who consistently graded CACT_MC_no bone_ not only as significantly better than CACT_Org_ concerning overall image quality but also judged its effect on motion artifacts higher than the more experienced readers and would have repeated more than half of the CACTs. Even though less experienced readers seemed to benefit more greatly from the algorithm, this is also true for more seasoned radiologists, as all intraclass correlations demonstrated good to excellent values. With the aforementioned post-processing steps taking up 5–7 min of time and being confined to a defined workspace, an automated bone segmentation would ultimately be required to organically integrate it into clinical routine and to reduce both manual interaction and reconstruction times.

### Limitations

This was a retrospective study and as only severely flawed CACTs were to be included, there was a relatively low number of suitable studies to choose from. A larger study population with a prospective, multi-centric study design and datasets from different angiographic systems would be beneficial in further analysing the motion correction algorithm’s effect. Thus, we are in the planning stages of a prospective study with patients receiving both an arterial and parenchymal CACT for selective internal radiation therapy (mprSIRT). Furthermore, up to now, no gold standard for the evaluation of CACTs exists, which is why we adapted previously published scoring features in combination with expert consensus criteria.

## Conclusion

The application of the motion correction algorithm in CACTs during TACE with limited diagnostic value leads to a significant improvement in image quality. IQ increased especially in cases of a high artery-to-parenchyma ratio and has the potential to limit radiation exposure due to repeated CACT.

## Data Availability

N.A.

## Abbreviations

(CA)CT: (C-arm) Computed tomography
DSA: Digital subtraction angiography
HCC: Hepatocellular carcinoma
HU: Hounsfield Units
IQ: Image quality
MC: Motion correction
MIP: Maximum intensity projection
Org: Original
s: Seconds
SD: Standard deviation
TACE: Transarterial chemoembolization

## References

[1] Angle JF (2013). Cone-beam CT: vascular applications. Tech Vasc Interv Radiol.

[2] Hinrichs JB, Marquardt S, von Falck C (2016). Comparison of C-arm computed tomography and digital subtraction angiography in patients with chronic thromboembolic pulmonary hypertension. Cardiovasc Intervent Radiol.

[3] Hinrichs JB, Renne J, Hoeper MM (2016). Balloon pulmonary angioplasty: applicability of C-arm CT for procedure guidance. Eur Radiol.

[4] Tacher V, Bhagat N, Rao PV (2013). Image quality improvements in C-arm CT (CACT) for liver oncology applications: preliminary study in rabbits. Minim Invasive Ther Allied Technol.

[5] Tacher V, Radaelli A, Lin M, Geschwind JF (2015). How I do it: cone- beam CT during transarterial chemoembolization for liver cancer. Radiol.

[6] Pung L, Ahmad M, Mueller K (2017). The Role of Cone-Beam CT in Transcatheter Arterial Chemoembolization for Hepatocellular Carcinoma: A Systematic Review and Meta-analysis. J Vasc Interv Radiol.

[7] Meyer BC, Frericks BB, Albrecht T, Wolf KJ, Wacker FK (2007). Contrast-enhanced abdominal angiographic CT for intra-abdom- inal tumor embolization: a new tool for vessel and soft tissue visualization. Cardiovasc Intervent Radiol.

[8] Meyer BC, Frericks BB, Voges M (2008). Visualization of hypervascular liver lesions during TACE: comparison of angio- graphic C-arm CT and MDCT. AJR Am J Roentgenol.

[9] Meyer BC, Witschel M, Frericks BB (2009). The value of combined soft-tissue and vessel visualisation before transarterial chemoembolisation of the liver using C-arm computed tomog- raphy. Eur Radiol.

[10] Syha R, Grozinger G, Grosse U (2015). C-arm computed tomography parenchymal blood volume measurement in evaluation of hepatocellular carcinoma before transarterial chemoembolization with drug eluting beads. Cancer Imaging.

[11] Wacker FK, Meissner OA (2009). Meyer BC [C-arm CT for planning and guidance of extrahepatic embolizations]. Radiologe.

[12] Burrel M, Reig M, Forner A (2012). Survival of patients with hepatocellular carcinoma treated by transarterial chemoembolization (TACE) using drug eluting beads. Implications for clinical practice and trial design. J Hepatol.

[13] European Association for the Study of the Liver (2018). EASL clinical practice guidelines: management of hepatocellular carcinoma. J Hepatol.

[14] Maschke SK, Hinrichs JB, Renne J (2019). C-arm computed tomography (CACT)-guided balloon pulmonary angioplasty (BPA): evaluation of patient safety and peri- and post-procedural complications. Eur Radiol.

[15] Bagla S, Rholl KS, Sterling KM (2013). Utility of cone-beam CT imaging in prostatic artery embolization. J Vasc Interv Radiol.

[16] Minami Y, Yagyu Y, Murakami T, Kudo M (2014). Tracking navigation imaging of transcatheter arterial chemoembolization for hepato- cellular carcinoma using three-dimensional cone-beam CT angiography. Liver Cancer.

[17] Miyayama S, Yamashiro M, Hashimoto M (2013). Identification of small hepatocellular carcinoma and tumor-feeding branches with cone-beam CT guidance technology during transcatheter arterial chemoembolization. J Vasc Interv Radiol.

[18] Iwazawa J, Ohue S, Mitani T (2009). Identifying feeding arteries during TACE of hepatic tumors: comparison of C-arm CT and digital subtraction angiography. AJR Am J Roentgenol.

[19] Rohkohl C, Lauritsch G, Biller L, Hornegger J (2010). ECG-gated interventional cardiac reconstruction for non-periodic motion. Med Image Comput Comput Assist Interv.

[20] Rohkohl C, Lauritsch G, Prummer M, Hornegger J (2009). Interven- tional 4-D motion estimation and reconstruction of cardiac vas- culature without motion periodicity assumption. Med Image Comput Comput Assist Interv.

[21] Schultz CJ, Lauritsch G, Van Mieghem N (2015). Rotational angiography with motion compensation: first-in-man use for the 3D evaluation of transcatheter valve prostheses. EuroInterven- tion.

[22] Becker LS, Gutberlet M, Maschke SK (2021). Evaluation of a motion correction algorithm for C-Arm computed tomography acquired during transarterial chemoembolization. Cardiovasc Intervent Radiol.

[23] Maschke SK, Werncke T, Becker LS (2020). The value of C-arm computed tomography in addition to conventional digital subtraction angiography in the diagnostic work-up of patients with suspected chronic thromboembolic pulmonary hypertension: an update of 300 patients. Acad. Radiol..

[24] Cicchetti DV (1994). Guidelines, criteria and rules of thumb for evaluating normed and standardized assessment instruments in psychology. Psychol Assess.

[25] Lee IJ, Chung JW, Yin YH (2014). Cone-beam CT hepatic arteriography in chemoembolization for hepatocellular carcinoma: angiographic image quality and its determining factors. J Vasc Interv Radiol..

